# Prediction of non-muscle invasive bladder cancer outcomes assessed by innovative multimarker prognostic models

**DOI:** 10.1186/s12885-016-2361-7

**Published:** 2016-06-03

**Authors:** E. López de Maturana, A. Picornell, A. Masson-Lecomte, M. Kogevinas, M. Márquez, A. Carrato, A. Tardón, J. Lloreta, M. García-Closas, D. Silverman, N. Rothman, S. Chanock, F. X. Real, M. E. Goddard, N. Malats, On behalf of the SBC/EPICURO Study Investigators

**Affiliations:** Genetic and Molecular Epidemiology Group, Spanish National Cancer Research Centre (CNIO), C/Melchor Fernández, Almagro, 3, 28029 Madrid Spain; Centre for Research in Environmental Epidemiology (CREAL), Parc de Salut Mar, Barcelona, Spain; Servicio de Oncología, Hospital Universitario Ramon y Cajal, Madrid, and Servicio de Oncología, Hospital Universitario de Elche, Elche, Spain; Department of Preventive Medicine Universidad de Oviedo, Oviedo, Spain; Parc de Salut Mar and Departament of Pathology, Hospital del Mar - IMAS, Barcelona, Spain; Division of Genetics and Epidemiology, Institute of Cancer Research, London, UK; Division of Cancer Epidemiology and Genetics, National Cancer Institute, Department of Health and Human Services, Bethesda, Maryland, USA; Epithelial Carcinogenesis Group, Spanish National Cancer Research Centre (CNIO), Madrid, and Departament de Ciències Experimentals i de la Salut, Universitat Pompeu Fabra, Barcelona, Spain; Biosciences Research Division, Department of Environment and Primary Industries, Agribio, and Department of Food and Agricultural Systems, University of Melbourne, Melbourne, Australia; CIBERESP, Madrid, Spain

**Keywords:** Multimarker models, Bayesian statistical learning method, Bayesian regression, Bayesian LASSO, AUC-ROC, Determination coefficient, heritability, Bladder cancer outcome, Prognosis, Recurrence, Progression, Genome-wide common SNP, Illumina Infinium HumanHap 1 M array, Predictive ability

## Abstract

**Background:**

We adapted Bayesian statistical learning strategies to the prognosis field to investigate if genome-wide common SNP improve the prediction ability of clinico-pathological prognosticators and applied it to non-muscle invasive bladder cancer (NMIBC) patients.

**Methods:**

Adapted Bayesian sequential threshold models in combination with LASSO were applied to consider the time-to-event and the censoring nature of data. We studied 822 NMIBC patients followed-up >10 years. The study outcomes were time-to-first-recurrence and time-to-progression. The predictive ability of the models including up to 171,304 SNP and/or 6 clinico-pathological prognosticators was evaluated using AUC-ROC and determination coefficient.

**Results:**

Clinico-pathological prognosticators explained a larger proportion of the time-to-first-recurrence (3.1 %) and time-to-progression (5.4 %) phenotypic variances than SNPs (1 and 0.01 %, respectively). Adding SNPs to the clinico-pathological-parameters model slightly improved the prediction of time-to-first-recurrence (up to 4 %). The prediction of time-to-progression using both clinico-pathological prognosticators and SNP did not improve. Heritability (*ĥ*^2^) of both outcomes was <1 % in NMIBC.

**Conclusions:**

We adapted a Bayesian statistical learning method to deal with a large number of parameters in prognostic studies. Common SNPs showed a limited role in predicting NMIBC outcomes yielding a very low heritability for both outcomes. We report for the first time a heritability estimate for a disease outcome. Our method can be extended to other disease models.

**Electronic supplementary material:**

The online version of this article (doi:10.1186/s12885-016-2361-7) contains supplementary material, which is available to authorized users.

## Background

Urothelial bladder cancer (UBC) is among the most common malignant tumors of the urological system and one of the most prevalent cancers due to its chronic nature [1]. As a consequence, it poses an enormous burden on health care systems [2].

UBC also represents a paradigm of heterogeneous diseases with respect to its phenotype and prognosis. Approximately, 75 % of newly diagnosed UBCs do not invade the muscle (non-muscle invasive bladder cancer, NMIBC) at the time of diagnosis. Most of these cancers remain stable over the time after a transurethral resection (TUR); a high proportion relapse without invading the muscle (recurrence) while a lower proportion progress as a muscle invasive bladder cancer (MIBC). Based on tumor characteristics, mainly stage and grade, NMIBC are subsequently classified as “low risk” (LR) and “high risk” (HiR) of progression [3].

Current prognostic tools for NMIBC are based on well-known clinico-pathological prognosticators such as pathological grade and stage, number and size of tumours, and presence of carcinoma in situ [3, 4]. However, these factors do not have enough discriminative ability to predict, at the patient level, the risk of recurrence and progression [5]. An accurate estimation of the outcome risk in the individual patient would help identifying the most appropriate therapy to avoid tumor progression and, hopefully reducing the number of follow-up cystoscopies in patients at low risk [6].

There is a growing evidence for a role of germline genetic polymorphisms in cancer risk and prognosis, UBC being a paradigm [7, 8]. However, the individual effect of the genetic variants is expected to be small and they may not be medically actionable. Multimarker analyses have been shown to capture a much higher percentage of the genetic variance than individual markers which passed the significant threshold in GWAS [9–11].

Our objective was to investigate whether genome-wide common SNP profiles are able to predict the risk of recurrence and progression in NMIBC patients and to estimate how much they contribute to these predictions when combined with clinico-pathological prognosticators. To this end, we adapted Bayesian statistical learning strategies to be applied to the human prognosis field for the first time.

## Methods

### Study population

This study was performed in patients with primary UBC included in the Spanish Bladder cancer (SBC)/EPICURO Study. Cases were recruited in 18 hospitals and followed up >10 years after diagnosis. A total of 1,105 patients had their diagnosis confirmed through a pathological review conducted by a panel of experts. Trained monitors collected detailed data on clinico-pathological prognosticators from clinical charts and followed the patients up prospectively through the participating hospitals and direct telephone interviews.

In this study, we focused on patients with a primary diagnosis of NMIBC (*N* = 995). Two endpoints were of interest: 1) Time-to-first-recurrence (TFR), defined as the reappearance of a NMIBC tumor following a previous negative follow-up cystoscopy, and 2) time-to-progression (TP), defined as the development of a muscle invasive tumor or a metastatic disease, or death because of UCB, after a previous diagnosis of NMIBC. Patients who did not present any event until the end of study, those lost of follow up and those who died from other causes were considered as censored either at last medical visit or at death.

Patients who underwent to a cystectomy were not considered in the analyses of TFR. A final number of 810 and 822 cases with NMIBC were available for the analyses of TFR and TP, respectively: 284 were HiR tumors (Ta high grade, T1 high grade, carcinoma in situ (CIS) and T1 low grade tumors) and 538 LR tumors (those presenting papillary UBC of low malignant potential or Ta low-grade papillary UBC according to the 2004 WHO classification).

### Genotyping and quality control

Genotyping was performed as described in ^12^ and provided calls for 1,072,820 SNP genotypes. We excluded SNPs in sex chromosomes, those with a low genotyping rate (<95 %) and MAF < 0.02 in NMIBC.

Stringent LD pruning (*r*^*2*^ < 0.2) was applied to reduce the number of markers, prioritizing those with less missing data. In addition, SNPs found significant in a previous prognostic study were considered here [11]. The final numbers of assessed SNPs for TFR and TP were 171,295 and 171,304, respectively, providing a good coverage of the genome. Missing genotypes were imputed using the package randomForest in R [12].

### Statistical model

We used a sequential threshold model [13] to analyze time-to-event data. This approach was previously applied in quantitative genetics [13–15], although till present it has not been applied in a human genomic study. This model assumes that for an observation of a patient to be present at a given period of time, he/she must have survived through all previous time periods. Thus, the probability of not presenting the event of interest until interval *k*, conditional on the event that the *k*-th interval has been reached, is given by:

$$ \Pr \left({y}_i=k\Big|{y}_i\ge k-1,\boldsymbol{\upgamma}, \boldsymbol{\upbeta} \right)=\varPhi \left(\frac{\gamma_l-\mathbf{X}\hbox{'}\boldsymbol{\upbeta}}{\sigma_e}\right), $$

where **γ** corresponds to unordered cutoff points corresponding to each time interval, **X** corresponds to the incidence matrix of effects (**β**) affecting the liability to survive to the next interval given that the present interval has been reached. Residual variance (*σ*_*e*_^2^) was fixed to 1 to ensure identifiability of the parameters [16].

**Fig. 1:**
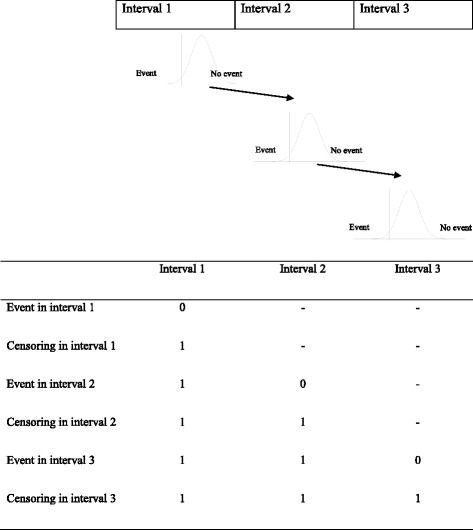
Data censoring in each defined interval according to the presence/absence of event when a sequential threshold model is applied

**Fig. 2:**
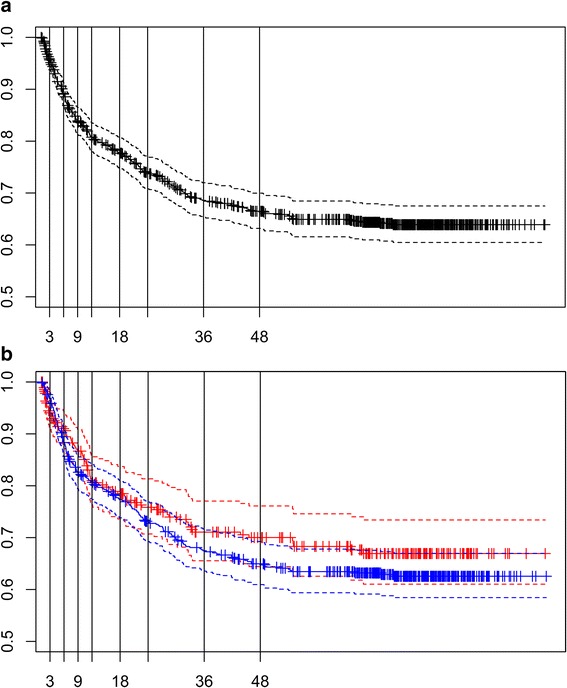
Survival function (solid line) and 95 % CI (dotted lines) of the time to recurrence (TFR) for the whole series (A) and according to the group of risk (B: HiR in red and LR in blue). Vertical lines separate the 9 time intervals considered for this outcome

**Fig. 3:**
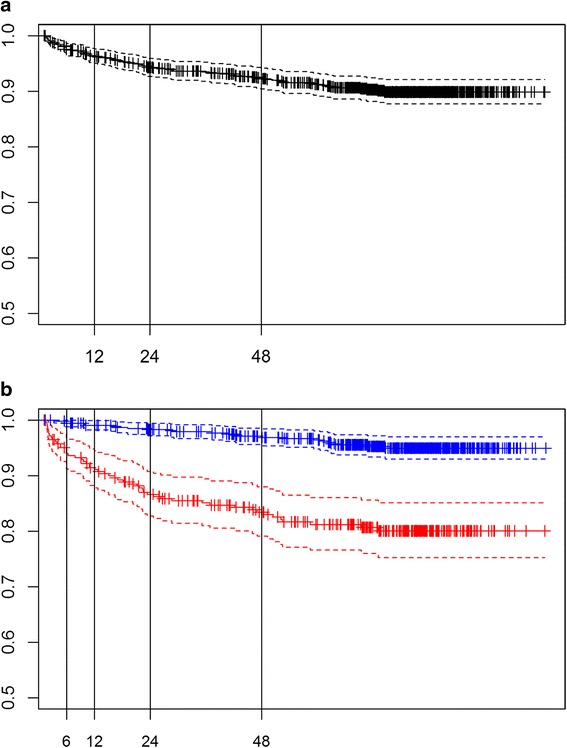
Survival function (solid line) and 95 % CI (dotted lines) of the time to progression (TP) for the whole series (**a**) and according to the group of risk (**b**: HiR in red and LR in blue). Vertical lines separate the 9 time intervals considered for this outcome

Patients were classified as censored or uncensored in each time interval considered for each event as displayed in Fig. 1. We divided the follow-up time for TFR and TP in 9 and 4 intervals, respectively, according to the survival functions for each event (see Figs. 2a and 3a). The analysis of TP was further stratified according to the tumor risk group (LR and HiR, see Fig. 3b). For these subgroup analyses the number of intervals was lower.

Three models were used in the analyses of each outcome: (1) Model including the clinico-pathological prognosticators only, (2) model including the SNP data only, and (3) model including both clinico-pathological prognosticators &SNP data. As for the first model, a Bayesian regression was used (see Additional file 1: Table S1). Further information of the model building is in Additional file 2: Supplementary methods. Regarding the second model, a Bayesian LASSO [17] was applied to analyze the predictive ability of common SNPs (see Additional file 2: Supplementary methods for further details). Finally, for the full model, a Bayesian regression coupled with LASSO [18, 19] was used. Priors and fully conditional distributions for both SNP and clinico-pathological prognosticators are described in Additional file 2: Supplementary methods.

### Evaluation of the predictive ability

The predictive ability of each model in the whole cohort was evaluated through a 10 fold cross-validation (CV) [20]. When patients were stratified as HiR/LR for the TP analyses, a 2-fold CV procedure was performed instead, due to the low number of events. We measured the predictive ability of each model using two statistics: 1) the area under the ROC (AUC), generated with the ROCR package for R (www.r-project.org), and the determination coefficient on the liability scale (*R*_*probit*_^2^), which is the proportion of the total variance explained by predictors in the testing set on the probit liability scale [21]:

$$ {R}_{probit}^2=\frac{\operatorname{var}\left({\mathbf{X}}_{test}\widehat{\boldsymbol{\upbeta}}\right)}{\operatorname{var}\left({\mathbf{X}}_{test}\widehat{\boldsymbol{\upbeta}}\right)+{\sigma}_e^2} $$

## Results

Additional file 1: Table S2 provides the number of censored patients and events in each time interval according to the outcome of interest (TFR and TP).

### Time to first recurrence

33 % of the patients with a primary NMIBC suffered a recurrence of the primary tumor (first recurrence). Fifty percent of patients presented the first recurrence during the first year and in most cases (94 %), the first recurrence was diagnosed during the first 4 years of follow up. Fifty-two percent of the NMIBC patients were censored at the end of the follow-up.

Table 1 and Additional file 1: Table S3 show the averaged AUC and *R*_*probit*_^2^ obtained after the 10 fold CV analyses with the three models. The model including clinico-pathological prognosticators had an averaged AUC of 0.62. Model including only SNPs classified slightly better than random (AUC = 0.55). The joint model did not perform better (AUC = 0.61).

**Table 1:** Averaged area under the ROC curve (AUC) and coefficient of determination (*R*_*probit*_^2^), as well standard deviations (between parenthesis), obtained from the testing sets in the 10 fold-crossvalidation analyses of time to first recurrence (TFR) and time to progression in the whole (TP), high risk (TPHiR) and low risk (TPLR) cohorts

Model	Criterion	TFR	TP	TPHiR	TPLR
		Whole series	Whole series	HiR tumors	LR tumors
CPP	AUC	0.62 (0.05)	0.76 (0.09)	0.57 (0.04)	0.45 (0.02)
	*R*_*probit*_^2^	0.031 (0.004)	0.054 (0.013)	0.151 (0.013)	0.0358 (0.0094)
SNPs	AUC	0.55 (0.02)	0.58 (0.09)	0.56 (0.01)	0.55 (0.01)
	*R*_*probit*_^2^	0.010 (0.001)	0.001 (0.000)	0.009 (0.002)	0.0005 (0.0002)
CPP&SNPs	AUC	0.61 (0.05)	0.76 (0.10)	0.57 (0.03)	0.47 (0.02)
	*R*_*probit*_^2^	0.041 (0.006)	0.050 (0.013)	0.155 (0.019)	0.0267 (0.0099)

*CPP* clinico-pathological prognosticators

When the predictive ability was evaluated using *R*_*probit*_^2^, the model combining clinico-pathological prognosticators &SNPs performed the best, capturing 4 % of the phenotypic variance on the liability scale. The predictive abilities for the clinico-pathological prognosticators and the SNP models were 3 and 1 %, respectively; the latter being the first heritability estimate (*ĥ*^2^) for TFR in NMIBC reported so far.

### Time to first progression

#### Whole cohort

Nine percent of the patients with a primary NMIBC suffered of a tumor progression during the follow-up. Fifty percent of the patients were diagnosed during the first two years and most of them (89 %) were diagnosed during the first 5 years (see Additional file 1: Table S2). Seventy five percent of the patients did not show any progression at the end of the follow-up period (>10 year). Table 1 and Additional file 1: Table S4 show the AUC and *R*_*probit*_^2^ after the 10 CV analyses for TP. The model including clinico-pathological prognosticators had an averaged AUC of 0.76, a much higher value than the model with SNPs only (AUC = 0.58). Adding SNPs to clinico-pathological prognosticators did not increase their individual classification performance (AUC = 0.76). Clinico-pathological prognosticators explained 5.4 % of the phenotypic variance on the liability scale. Surprisingly, SNP explained only 0.1 % of the variance. Adding SNPs to the clinico-pathological prognosticators worsened the *R*_*probit*_^2^ of the model (Table 1).

#### Patients at HiR

The majority (~70 %) of patients showed a progression during the first two years of follow-up and 75 % of them finished the follow-up without any progression (Additional file 1: Table S2). Table 1 and Additional file 1: Table S5 show the AUC and *R*_*probit*_^2^ of the three models evaluated. The model including only clinico-pathological prognosticators classified the patients according to the TP similarly to the model including only SNPs (0.57 vs. 0.56, respectively). The model with the best *R*_*probit*_^2^ for progression at HiR was the one considering clinico-pathological prognosticators (*R*_*probit*_^2^ = 0.151). Including only common SNPs explained <1 % of the phenotypic variance of the cohort at HiR. Adding them to the clinico-pathological prognosticators increased their predictive ability by 2.6 % (*R*_*probit*_^2^ = 0.155).

#### Patients at LR

Only 24 patients showed a progression during the follow-up (<5 %). Two thirds of those patients were diagnosed during the first 2 years of follow-up. Table 1 and Additional file 1: Table S6 present the AUC and *R*_*probit*_^2^ of the three models corresponding to the 2 fold-CV procedure. The model including clinico-pathological prognosticators poorly categorized LG-NMIBC patients according to their progression status (AUC = 0.45). By including age at diagnosis we obtained a better classification (AUC = 0.68). The SNP model classified the patients slightly better than random (AUC = 0.55). The best *R*_*probit*_^2^ was found for the model including only clinico-pathological prognosticators (0.0358). Adding SNPs to latter model worsened its *R*_*probit*_^2^ (0.0267).

## Discussion

Here we present a high dimensional model considering the time-to-event nature of the information and censored data enabling to accommodate a large number of variables in a relatively small number of individuals. To our knowledge, this is the first time that such a model is applied in the clinical and genetic epidemiology fields. More specifically, we have applied it to study the predictive ability of prognostic models for NMIBC patients.

The major goal in managing NMIBC patients is to prevent tumor relapse, this including both the high number of recurrences and the progression to MIBC. To this end, treatment needs to be tailored according to the aggressiveness of the disease. Therefore, accurate prognostic models are crucial. Currently, there are no validated prognostic molecular biomarkers to guide the clinical management of patients [22, 23] and the therapeutic decisions are still based on risk tables only including clinico-pathological prognosticators [3]. Here we have investigated the potential clinical utility of inherited genetic markers (SNP profiles) based on their robustness and precise measurements as well as on their time-independent nature in comparison to serological and histological markers. To this end we have assessed the ability to improve TFR and TP risk stratification in NMIBC patients of genome-wide common SNPs profiles. We have also evaluated the performance of well-known clinico-pathological prognosticators and how much the whole genome approach improved their performance to better classify patients.

Regarding the classification performance of clinico-pathological prognosticators alone, our sequential threshold models for both TFR and TP got similar estimates to those obtained previously by us with a Cox proportional hazard regression analysis [11]. Discrimination of patients according to the risk of TFR using clinico-pathological prognosticators was poorer than previously reported by Hernandez et al [24] (0.62 vs. 0.75), although better than that reported by Vedder et al [25] in a large cohort including ours. Nevertheless, it is worth noting that the definition of the outcome differs (recurrence vs. first recurrence) between our and these studies [24, 25]. Regarding TP outcome, our clinico-pathological prognosticators model classified the patients better than in Hernandez et al [24] (0.76 vs. 0.54) and than in a Danish cohort using both EORTC (0.76 vs. 0.72) and CUETO (0.76 vs. 0.74) scores [25]. However, it performed worse than in a Dutch cohort using the same classifiers: EORTC (0.76 vs. 0.81 and 0.77) and CUETO scores (0.76 vs. 0.82 and 0.81) [25].

The prediction ability of clinico-pathological prognosticators depends on the outcome. They clearly perform better in predicting TP than TFR, both in terms of classification (AUC, 0.76 vs. 0.62) and proportion of the explained variance (*R*_*probit*_^2^, 5.4 % vs. 3.1 %). Their lower performance when predicting TFR could be due to the dependence of factors other than biological explanations such as the potential incomplete resection of the tumor during the TURB and the tumour cell reimplantation on first tumour recurrence [23], factors that are difficult to be assessed and therefore are not accounted for in the model. When the patients were stratified according to their risk status, clinico-pathological prognosticators explained a larger proportion of the phenotypic variance (~15 %) in the HiR group than in the LR NMIBC, probably because these factors were specifically selected to identify patients with HiR tumors with a high potential of progression. However, the overall classification performance of HiR NMIBC patients was poorer (AUC = 0.57) than in the whole cohort. While the discriminatory ability of clinical-pathological parameters for both NMIBC outcomes is valuable, there is room for improvement. More accurate discriminatory models would better select patients for aggressive treatment as well as would avoid unnecessary treatments towards a better patient management. This justifies the search of further prognostic factors, among them tumour molecular alteration and inherited variation markers [3, 26, 27].

Our results showed that common genome-wide SNPs similarly, though poorly, classified patients regarding both TFR and TP in the whole series and in the HiR and LR subcohorts, AUCs ranging from 0.55 to 0.58. Adding SNP to the models did not improve the classification performance of clinico-pathological prognosticators although improvements of *R*_*probit*_^2^ were achieved for TFR (3–4) and TP in the HiR cohort (15.1 - 15.5 %). Surprisingly, adding SNP to clinico-pathological prognosticators worsened the percentage of phenotypic variance (*R*_*probit*_^2^) explained by the model with clinico-pathological prognosticators only by 7 and 25 % when predicting TP in the whole and the LR-NMIBC cohorts, respectively. The little improvement or even deterioration in terms of *R*_*probit*_^2^ could be explained by a correlation between the prediction of clinico-pathological prognosticators and that of SNPs. To confirm this, we calculated the *R*_*probit*_^2^ of a model with $\mathbf{X}\widehat{\boldsymbol{\upbeta}}$obtained from clinico-pathological prognosticators only as dependent variable and the SNPs as independent variables (see Tables 2 and Additional file 1: Table S6). The proportion of the clinico-pathological prognosticators prediction variances of TFR and TP explained by SNPs was larger than that of the TFR and TP phenotypic variances. The calculation of *R*_*probit*_^2^ allowed us to report the first *ĥ*^2^ for TFR and TP in the whole series and in the HiR and LR subcohorts. The largest *ĥ*^2^ corresponded to TFR (1 %) and to TP of patients at HiR (1 %), although they may be underestimated because of the sample size and the limitation on the number of SNPs included in the model [28]. All the above explains the small or nil contribution of the SNPs to the predictive ability of clinico-pathological prognosticators of the phenotypes of interest. The poor predictive ability of common SNPs in NMIBC prognosis is in line with a previous study reporting low GWAS risk predictive values for UBC [19], as well as with those obtained in studies predicting risk for other neoplasms, such as breast cancer [29, 30]. The different results obtained with AUC and *R*_*probit*_^2^ can be explained by the different scales in which the predictions are expressed (observable for AUC and liability for *R*_*probit*_^2^), their non-monotonic relationship, and the lower number of events, especially when the individuals were stratified.

**Table 2:** Estimates of the determination coefficient (*R*_*probit*_^2^) measuring the proportion of variance of the liability to first recurrence (TFR) and progression (TP) risks in whole, high risk (TPHiR) and low risk (TPLR) cohorts of the clinicopathological prognosticators explained by the common SNPs

	TFR	TP	TPHiR	TPLR
	Whole series	Whole series	HiR tumors	LR tumors
*R*_*probit*_^2^	0.0260	0.0165	0.0025	0.0066

While this is one of the largest and well-characterized NMIBC cohort worldwide, the restricted sample size in the subgroup analyses is one of the limitations we face here because the small number of events limits the prediction accuracy of the genomic profile achieved with the SNPs. This is even clearer when patients were further stratified as LR-NMIBC. Although increasing sample size of the study would be desirable, heterogeneity across studies regarding patient recruitment, pathological classifications applied, and treatment or patient management would increase random misclassification and, therefore, would dilute estimates. While we conducted a genome-wide exploration, the models did not include all genotyped SNPs (1 million) but a subset that were filtered by a restrict LD. When we applied a less restrictive LD threshold (*r*^*2*^ < 0.8) and considered a larger number of common SNPs neither the classification performance nor the percentage of the phenotypic variance explained improved (results not shown). Including in the models both rare and structural variants may help in further characterizing and increase the precision of the predictive estimates. Application of other statistical modeling approaches could indeed yield improvements in the predictive power, for example by considering non-additive models that include epistatic interactions between SNPs or adding functional information in the model. Exploring the integration of other –omics data such as microRNAs, as well as considering possible interactions between treatment and variants could also help in this regard.

This study also presents several strengths as its population-based nature, detailed medical information, long follow-up, and centralized pathological review decreasing heterogeneity of the covariates stage and grade. The use of state-of-the art methodology applied here allowed to handle a highly dimensional problem and time-to-event data, as well as censoring. The application of such methodology allowed us to provide the first estimates of heritability for UBC outcomes.

## Conclusions

Here we provide the scientific community, for the first time, with a methodology to estimate the heritability and the prediction ability of multidimensional data in the prognosis field. By applying it to the UBC setting, we observed that the role of common SNPs is very limited in the prediction of risk of recurrence and progression in NMIBC. Future studies should explore whether the integration of other genetic variants, as well as their interaction among them and with treatment, contribute to build a more accurate predictive model allowing the final assessment of the translational potential of genetic inherited variants into the clinics.

## Appendix 1. Participating centers in the study

U.S. National Cancer Institute (NCI)

Institut Municipal d’Investigació Mèdica and Hospital del Mar

Centro Nacional de Investigaciones Oncológicas (CNIO)

Hospital Germans Tries i Pujol (Badalona, Barcelona)

Hospital de Sant Boi (Sant Boi, Barcelona)

Centre Hospitalari Parc Taulí (Sabadell, Barcelona)

Centre Hospitalari i Cardiològic (Manresa, Barcelona)

Hospital Universitario (La Laguna, Tenerife)

Hospital La Candelaria (Santa Cruz, Tenerife)

Hospital General Universitario de Elche

Universidad Miguel Hernández (Elche, Alicante)

Universidad de Oviedo (Oviedo, Asturias)

Hospital San Agustín (Avilés, Asturias)

Hospital Central Covadonga (Oviedo, Asturias)

Hospital Central General (Oviedo, Asturias)

Hospital de Cabueñes (Gijón, Asturias)

Hospital de Jove (Gijón, Asturias)

Hospital de Cruz Roja (Gijón, Asturias)

Hospital Alvarez-Buylla (Mieres, Asturias)

Hospital Jarrio (Coaña, Asturias)

Hospital Carmen y Severo Ochoa (Cangas, Asturias)

## Additional files

Additional file 1:
**Table S1.** Clinico-pathological variables included in the predictive models for time to first recurrence (TFR) and time to progression (TP). **Table S2.** Summary of censored patients and events (%) for each event in each time interval defined for the statistical analyses. **Table S3.** Area under the ROC curve (AUC) and coefficient of determination (*R*_*probit*_^2^) obtained for each testing set in the 10 fold-crossvalidation analyses of time to first recurrence. **Table S4.** Area under the ROC curve (AUC) and coefficient of determination (*R*_*probit*_^2^) obtained for each testing set in the 10 fold-crossvalidation analyses of time to progression. **Table S5.** Area under the ROC curve (AUC) and coefficient of determination (*R*_*probit*_^2^) obtained for each testing set in the 2 fold-crossvalidation analyses of time to progression in patients at high risk. **Table S6.** Area under the ROC curve (AUC) and coefficient of determination (*R*_*probit*_^2^) obtained for each testing set in the 2 fold-crossvalidation analyses of time to progression in patients at low risk. **Table S7.** Coefficient of determination (*R*_*probit*_^2^) obtained for each testing set in the 10 fold-crossvalidation analyses of time to first recurrence (TFR), time to progression (TP) in the whole cohort, and time to progression (TP) in the high and low risk cohorts (TPHiR and TPLR). (DOC 113 kb) Additional file 2: Supplemental Methods. Model including non-genetic variables. (DOC 55 kb)

## Abbreviations

AUC-ROC: area under the receiving operating curve
CiS: carcinoma in situ
GWAS: genome wide association study
HiR: high risk
LASSO: least absolute shrinkage and selection operator
LD: linkage disequilibrium
LR: low risk
MIBC: muscle invasive bladder cancer
NMIBC: non-muscle invasive bladder cancer
SBC: Spanish Bladder Cancer
SNP: single nucleotide polymorphism
TFR: time-to-first-recurrence
TP: time-to-progression
TURB: transurethral resection of the bladder
UBC: urothelial bladder cancer
WHO: World health organization
