# Supplementary material for: Prediction of non-muscle invasive bladder cancer outcomes assessed by innovative multimarker prognostic models
Source: BMC Cancer. 2016 Jun 3;16:351. doi: 10.1186/s12885-016-2361-7 (PMC4893282; doi:10.1186/s12885-016-2361-7)
Supplement: Additional file 1: — Table S1. Clinico-pathological variables included in the predictive models for time to first recurrence (TFR) and time to progression (TP). Table S2. Summary of censored patients and events (%) for each event in each time interval defined for the statistical analyses. Table S3. Area under the ROC curve (AUC) and coefficient of determination (R probit2) obtained for each testing set in the 10 fold-crossvalidation analyses of time to first recurrence. Table S4. Area under the ROC curve (AUC) and coefficient of determination (R probit2) obtained for each testing set in the 10 fold-crossvalidation analyses of time to progression. Table S5. Area under the ROC curve (AUC) and coefficient of determination (R probit2) obtained for each testing set in the 2 fold-crossvalidation analyses of time to progression in patients at high risk. Table S6. Area under the ROC curve (AUC) and coefficient of determination (R probit2) obtained for each testing set in the 2 fold-crossvalidation analyses of time to progression in patients at low risk. Table S7. Coefficient of determination (R probit2) obtained for each testing set in the 10 fold-crossvalidation analyses of time to first recurrence (TFR), time to progression (TP) in the whole cohort, and time to progression (TP) in the high and low risk cohorts (TPHiR and TPLR). (DOC 113 kb) [file 12885_2016_2361_MOESM1_ESM.doc]

**Supplemental Material**

**Table S1.** Clinico-pathological variables included in the predictive models for time to first recurrence (TFR) and time to progression (TP).

| TFR | Area + gender + # of tumours^a^ + TSG^b^ + tumour size^c^ + treatment^d^ |
| --- | --- |
| TP | Area + age + # of tumours^a^ + TSG^b^ + # of recurrences^e^ + treatment^f^ |

^a^ 1) One tumour, 2) >1, and 3) missing data.

^b^ TSG (tumour stage and grade): 1) PUNLMP+TaG1, 2) TaG2, 3) TaG3, 4) T1G2, 5) T1G3 and 6) TiG2+TiG3.

^c^ 1) <3 cm, 2) >3 cm and 3) missing data.

^d^ 1) Trans urethral resection (TURB) alone, 2) TURB+intravesical chemotherapy, 3) TURB +Bacillus Calmette Guerin (BCG), 4) TURB + intravesical chemotherapy + BCG, 5) Other treatments.

^e^ 1) 1, 2) 2, 3) 3, and 4) >3 previous recurrences.

^f^ 5 categories as in ^d^ plus TURB + radical cystectomy.

Table S2. Summary of censored patients and events (%) for each event in each time interval defined for the statistical analyses.

| **Time to recurrence** | **Whole set** | **Intervals** | **<=3** | **4-6** | **7-9** | **10-12** | **13-18** | **19-24** | **25-36** | **37-48** | **>48** |
| --- | --- | --- | --- | --- | --- | --- | --- | --- | --- | --- | --- |
|  |  | Events | 35 | 52 | 40 | 24 | 23 | 28 | 37 | 14 | 15 |
|  |  | Censored^a^ | 14 | 13 | 8 | 12 | 14 | 16 | 22 | 24 | 419 |
|  |  | Passed^b^ | 761 | 696 | 648 | 612 | 575 | 531 | 472 | 434 | - |
| **Time to progression** | **Whole set** | **Intervals** | **<=12** | | | | **13-24** | | **25-48** | | **>48** |
|  |  | Events | 30 | | | | 16 | | 15 | | 15 |
|  |  | Censored^c^ | 21 | | | | 23 | | 63 | | 639 |
|  |  | Passed^b^ | 771 | | | | 732 | | 654 | | - |
| **Time to progression** | **High risk** | **Intervals** | **<=6** | | **7-12** | | **13-24** | | **>24** | | |
|  |  | Events | 17 | | 8 | | 9 | | 15 | | |
|  |  | Censored^c^ | 5 | | 3 | | 12 | | 215 | | |
|  |  | Passed^b^ | 262 | | 251 | | 230 | | - | | |
| **Time to progression** | **Low risk** | **Intervals** | **<=24** | | | | | | **25-48** | | **>48** |
|  |  | Events | 9 | | | | | | 7 | | 8 |
|  |  | Censored^c^ | 27 | | | | | | 35 | | 452 |
|  |  | Passed^b^ | 502 | | | | | | 452 | | - |

^a^ Include those who left the study; finished the follow-up period without having any recurrence, died due to any cause during the follow up or had a progression; ^b^ NMIBC patients who have data (event of interest or censored data) in posterior follow-up intervals; ^c^ dead due to other causes

Table S3. Area under the ROC curve (AUC) and coefficient of determination ($R_{probit}^{2}$) obtained for each testing set in the 10 fold-crossvalidation analyses of time to first recurrence.

| **Model** | **Criterion** | **set1** | **set2** | **set3** | **set4** | **set5** | **set6** | **set7** | **set8** | **set9** | **set10** |
| --- | --- | --- | --- | --- | --- | --- | --- | --- | --- | --- | --- |
| CP | AUC | 0.63 | 0.56 | 0.53 | 0.65 | 0.62 | 0.65 | 0.67 | 0.58 | 0.67 | 0.62 |
|  | $R_{probit}^{2}$ | 0.029 | 0.040 | 0.038 | 0.024 | 0.032 | 0.029 | 0.030 | 0.026 | 0.033 | 0.030 |
| SNPs | AUC | 0.54 | 0.52 | 0.53 | 0.53 | 0.57 | 0.56 | 0.58 | 0.54 | 0.56 | 0.55 |
|  | $R_{probit}^{2}$ | 0.010 | 0.010 | 0.011 | 0.010 | 0.010 | 0.010 | 0.010 | 0.012 | 0.011 | 0.010 |
| CP+SNPs | AUC | 0.62 | 0.55 | 0.54 | 0.65 | 0.62 | 0.63 | 0.67 | 0.55 | 0.65 | 0.58 |
|  | $R_{probit}^{2}$ | 0.037 | 0.050 | 0.053 | 0.030 | 0.42 | 0.038 | 0.042 | 0.039 | 0.041 | 0.039 |

Table S4. Area under the ROC curve (AUC) and coefficient of determination ($R_{probit}^{2}$) obtained for each testing set in the 10 fold-crossvalidation analyses of time to progression.

| **Model** | **Criterion** | **set1** | **set2** | **set3** | **set4** | **set5** | **set6** | **set7** | **set8** | **set9** | **set10** |
| --- | --- | --- | --- | --- | --- | --- | --- | --- | --- | --- | --- |
| CP | AUC | 0.63 | 0.74 | 0.73 | 0.68 | 0.79 | 0.81 | 0.84 | 0.88 | 0.84 | 0.62 |
|  | $R_{probit}^{2}$ | 0.076 | 0.081 | 0.050 | 0.048 | 0.050 | 0.040 | 0.058 | 0.048 | 0.047 | 0.046 |
| SNPs | AUC | 0.46 | 0.47 | 0.58 | 0.59 | 0.56 | 0.49 | 0.60 | 0.75 | 0.66 | 0.63 |
|  | $R_{probit}^{2}$ | 0.001 | 0.001 | 0.001 | 0.001 | 0.001 | 0.001 | 0.001 | 0.001 | 0.001 | 0.001 |
| CP+SNPs | AUC | 0.63 | 0.75 | 0.76 | 0.68 | 0.79 | 0.80 | 0.85 | 0.89 | 0.86 | 0.60 |
|  | $R_{probit}^{2}$ | 0.072 | 0.072 | 0.044 | 0.046 | 0.045 | 0.036 | 0.056 | 0.044 | 0.042 | 0.041 |

Table S5. Area under the ROC curve (AUC) and coefficient of determination ($R_{probit}^{2}$) obtained for each testing set in the 2 fold-crossvalidation analyses of time to progression in patients at high risk.

| **Model** | **Criterion** | **Set1** | **Set2** |
| --- | --- | --- | --- |
| CP | AUC | 0.55 | 0.60 |
|  | $R_{probit}^{2}$ | 0.160 | 0.141 |
| SNPs | AUC | 0.55 | 0.57 |
|  | $R_{probit}^{2}$ | 0.011 | 0.008 |
| CP+SNPs | AUC | 0.55 | 0.60 |
|  | $R_{probit}^{2}$ | 0.169 | 0.142 |

Table S6. Area under the ROC curve (AUC) and coefficient of determination ($R_{probit}^{2}$) obtained for each testing set in the 2 fold-crossvalidation analyses of time to progression in patients at low risk.

| **Model** | **Criterion** | **Set1** | **Set2** |
| --- | --- | --- | --- |
| CP | AUC | 0.47 | 0.44 |
|  | $R_{probit}^{2}$ | 0.0425 | 0.0292 |
| SNPs | AUC | 0.56 | 0.54 |
|  | $R_{probit}^{2}$ | 0.0003 | 0.0006 |
| CP+SNPs | AUC | 0.48 | 0.45 |
|  | $R_{probit}^{2}$ | 0.0337 | 0.0197 |

Table S7. Coefficient of determination ($R_{probit}^{2}$) obtained for each testing set in the 10 fold-crossvalidation analyses of time to first recurrence (TFR), time to progression (TP) in the whole cohort, and time to progression (TP) in the high and low risk cohorts (TPHiR and TPLR).

| **Model** | **set1** | **set2** | **set3** | **set4** | **set5** | **set6** | **set7** | **set8** | **set9** | **set10** |
| --- | --- | --- | --- | --- | --- | --- | --- | --- | --- | --- |
| TFR | 0.0400 | 0.0223 | 0.0322 | 0.0178 | 0.0209 | 0.0232 | 0.0357 | 0.0322 | 0.0206 | 0.0150 |
| TP | 0.0124 | 0.0142 | 0.0186 | 0.0205 | 0.0141 | 0.0175 | 0.0211 | 0.0127 | 0.0167 | 0.0177 |
| TPHiR | 0.0023 | 0.0027 | - | - | - | - | - | - | - | - |
| TPLR | 0.0062 | 0.0070 | - | - | - | - | - | - | - | - |
