# Supplementary material for: Prediction of non-muscle invasive bladder cancer outcomes assessed by innovative multimarker prognostic models
Source: BMC Cancer. 2016 Jun 3;16:351. doi: 10.1186/s12885-016-2361-7 (PMC4893282; doi:10.1186/s12885-016-2361-7)
Supplement: Additional file 2: — Supplemental Methods. Model including non-genetic variables. (DOC 55 kb) [file 12885_2016_2361_MOESM2_ESM.doc]

**Supplemental Methods**

*Model including non-genetic variables.*

The vector of non-genomic variable effects ($\mathbf{\beta}_{CPP}$) was assigned a flat prior. The fully conditional distributions of $\mathbf{\beta}_{CPP}$ were $N\left(\mathbf{X'X}^{-\mathbf{1}}\mathbf{X'l},\mathbf{X'X}^{-\mathbf{1}}\sigma_{e}^{2}\right)$. $\mathbf{l}$ is the vector containing the liabilities to a given outcome, **X** corresponds to the incidence matrix of effects ($\mathbf{\beta}_{CPP}$) and $\sigma_{e}^{2}$ is the residual variance, which was set to 1 for identification purposes.

*Model including only genomic information.* An exponential density (double exponential prior for marker effects, $\prod_{SNP=1}^{m} N\left(\beta_{SNP}\left|0,\tau_{SNP}^{2}\sigma_{e}^{2}\right.\right)\times\prod_{SNP=1}^{m} \exp\left(\tau_{SNP}^{2}\left|\lambda\right.\right)$) was used as prior. Parameter $\lambda$ in BL controls the shape of the prior distribution assigned to $\tau_{SNP}^{-2}$, assigning more density to small values of $\tau_{SNP}$ than to large ones, and follows, a priori, a Gamma distribution $G\left(\lambda^{2}\left|10,0.75\right.\right)$. The fully conditional distribution of SNP effects ($\mathbf{\beta}_{SNP}$) was multivariate normal with mean (covariance matrix) equal to the solution (inverse of coefficient matrix) of the system of equations $\left[\mathbf{X}^{i}\mathbf{X}\sigma_{e}^{-2}+Diag\left(\tau_{SNP}^{-2}\sigma_{e}^{-2}\right)'\right]\hat{\mathbf{\beta}}_{SNP}=\mathbf{X}^{'}\mathbf{y}\sigma_{e}^{-2}$. The fully conditional distributions of $\tau_{j}^{-2}$ were inverse Gaussians with mean $\mu_{SNP}=\left(\sigma_{e}\lambda/\left|\beta_{SNP}\right|\right)$ and scale parameter $\lambda^{2}$. The fully conditional posterior distribution of $\lambda^{2}$ is $Gamma\left(p+10,\frac{1}{2}\sum_{SNP=1}^{m} \tau_{SNP}^{2}+0.75\right)$.

*10-fold cross validation procedure.* It consisted in randomly dividing the patients codified as 0/1 into ten non-overlapping and approximately equally sized parts in each defined time interval to ensure a similar proportion of 0/1 in both training and testing sets. The statistical model was fitted by pooling nine-tenths of the data (the so-called ‘training set’), and the predictive ability was computed with the remaining one-tenth (the so-called ‘testing set’). This procedure was repeated 10 times, so that every fold was predicted.
